# Doping of ZnO inorganic-organic nanohybrids with metal elements

**DOI:** 10.1038/s41598-019-48497-3

**Published:** 2019-08-16

**Authors:** Y. Zhang, A. Apostoluk, C. Theron, T. Cornier, B. Canut, S. Daniele, B. Masenelli

**Affiliations:** 1 Institut des Sciences Appliquées de Lyon, INL - UMR 5270, Université de Lyon, INSA-Lyon, ECL, UCBL, CPE, CNRS, 69621 Villeurbanne, France; 2 Université Lyon 1, Université de Lyon, IRCE Lyon, CNRS, UMR 5256, 69626 Villeurbanne, France

**Keywords:** Nanoparticles, Nanoparticles

## Abstract

We present a general and in-depth study of the effect of dopants in hybrid inorganic/organic ZnO/PAA (polyacrylic acid) nanocomposites. These dopants vary as much by their ionic size, as by their electronic valence and some of them have been used in ZnO due to their known magnetic and/or optical properties. The chemical nature of the dopants controls their ability to incorporate into ZnO crystal lattice. Three concentrations (0.1%, 1% and 5%) of dopants were studied in order to compare the effect of the concentration with the results obtained previously in the literature. Our results confirm in the first place the trend observed in the literature, that increase in dopant concentration leads to quenching of visible luminescence for ZnO nanocrystals obtained by very different processes. However, the degradation of photoluminescence quantum yield (PL QY) is not inevitable in our nanocomposites. At low doping concentration for some dopants with a small or comparable ionic radius than Zn^2+^, PL QY can be maintained or even improved, making it possible to tune the visible emission spectrum between 2.17 eV and 2.46 eV. This opens up the prospect of synthesizing phosphors without rare earth for white LEDs, whose spectrum can be tuned to render warm or cold white light, by a chemical synthesis process with a low environmental impact.

## Introduction

The market of white-LED phosphors is dominated by rare-earth based technology. More specifically, the most common phosphor, absorbing blue light from InGaN LED and re-emitting yellow light, is YAG:Ce (yttrium aluminum garnet doped with Ce^3+^ ions). This technology is very efficient and presents phosphors with an internal quantum yield (the ratio of the number of emitted photons to the number of absorbed photons) second to none^1–3^. However, it relies on a critical supply chain and an ecologically unfriendly material industry (rare-earth industry). Therefore, there is a growing demand for alternative phosphors. One strategy is to harvest luminescence originating from intrinsic defects of selected materials. The emission from crystalline defects in ZnO nanomaterials is a prototype of this strategy. ZnO having a wide bandgap (3.34 eV at room temperature) is able to absorb near-UV light (from GaN LEDs for instance). Moreover, it exhibits many crystalline defects that can emit visible light, such as oxygen or zinc vacancies (V_O_ or V_Zn_), oxygen or zinc interstitials (O_i_ or Zn_i_) or combinations of these single defects^4–6^. Some of them are so efficient an emitter that they can be used as single photon sources^7–9^. These defects emit from the blue to the red, with distinct PL QY. The biggest issue is to control their concentration, nature and position in order to control the overall PL QY and the resulting emission color.

It is thus delicate to fine tune the color of the emitted light (in order to produce “cold” or “warm” white light) relying alone on the native defects present in ZnO nanocrystal since their positions in the bandgap are relatively set^10^. An alternative would be to modify the emitted light by introducing selected dopant atoms into ZnO lattice. Doping of ZnO has been tempted with many chemical elements, mostly with magnetic ones, to produce diluted magnetic semiconductors (DLS), but also with non-magnetic ones^11–21^. The effect of these dopants on the optical properties of doped ZnO is not definitely established, but there seems to be a tendency that doping quenches the UV and visible luminescence, most probably through the introduction of non-radiative levels in the bandgap^22^. On the contrary, certain dopants, such as Cu, are known to introduce peculiar radiative gap states^23^. Besides, the chemical nature of the dopant is not the only parameter to consider. The dopant concentration and its valence state are also to be taken into account. Finally, the quenching effect of dopants involuntarily lowers PL efficiency but to what extent is unknown. Some studies have reported PL QY of ZnO which can be altered (mostly improved) by doping, surface modification, excitation density and temperature^24–28^, however, no comparison of such alteration between different dopants has been reported so far. To sum up, the question about the possibility to tune the color of the emitted light, while maintaining PL QY, in doped ZnO nanostructures is not solved yet.

In this perspective, the present work investigates the effects of various dopants of different valence states and concentrations on the optical properties of hybrid polymer/ZnO nanomaterials. The hybrid polymer/ZnO nanostructures are produced by chemical synthesis route using the hydrolysis method, starting from diethyl zinc and precursors of the dopants in presence of a weak polymeric acid, namely polyacrylic acid (PAA). In a previous study^29^, we have shown that in the absence of dopants, this synthesis process can lead to highly efficient emitting structures, with PL QY higher than 20%, even reaching 70% and stable in time (over several weeks). We have focused on several distinct doping species, ranging from well-known dopants, such as Cu, to more prospective ones. These dopants differ in their precursor valence state from 1+ to 4+, as well as in their ionic radius. These two characteristics are bound to have a major effect on their incorporation and activation mechanisms and thus on the luminescence properties of the doped ZnO hybrid nanostructures. A quantitative study in PL QY of doped ZnO was also carried out in order to obtain a clear picture of how different dopants affect luminescence efficiency of ZnO nanoparticles. Ultimately, the addressed question is whether it is possible to tune the emission color thanks to the incorporation of dopants without degrading PL QY with respect to the undoped ZnO structure. This would open the door to the synthesis of phosphors for white LEDs enabling the control of LED emission from cold to warm white.

## Synthesis Protocol and Experimental Characterization

### Synthesis route

The synthesis protocol used in the present study reproduces the one used in our previous study, detailed in^29^. It is based on the hydrolysis of diethyl zinc (ZnEt_2_). 1.8 ml of a solution of ZnEt_2_ (15 wt%) in toluene is added dropwise in an aqueous solution containing 0.63 wt% of a weak polymeric acid, namely polyacrylic acid (PAA). The polymeric acid has a low molecular weight (2000 g.mol^−1^), since our previous study has shown that low molecular weight is beneficial to the synthesis of bright ZnO nanocrystals embedded in polymer mesospheres. During the synthesis, the mixture is vigorously stirred under inert atmosphere since ZnEt_2_ is pyrophoric. The ethane resulting from the hydrolysis is constantly evacuated via a bubbler.

Dopant salts are solubilized in the PAAH aqueous solution before adding ZnEt_2_. Three typical concentrations of precursors have been used, namely 0.1 wt%, 1 wt% and 5 wt%. We have selected twelve (one element Cr being introduced with two distinct precursors, namely CrCl_3_ and Cr(NO_3_)_3_) doping ions, to cover a broad range of ionic radii and valence states, as listed in Table 1. It should be noted that we have not verified by XPS or other means the actual valence state of the doping elements in the samples. When mentioning a state of valence, we are referring only to the state of valence of the metallic doping element in its precursor molecule. The majority of the selected doping elements have been investigated independently in the literature^11–21,30–35^. Our study therefore aims at rationalizing the study of their effect on the ZnO nanocrystal optical properties in the same condition of elaboration.

**Table 1:** List of the doping elements, sorted according to their ionic radius.

Element	Al^3+^	Fe^3+^	Fe^2+^	Cr^3+^	Co^2+^	Mn^2+^	Ni^2+^	Cu^2+^	Cu^+^	Ce^4+^	Bi^3+^	Ag^+^
Ionic radius (pm)	67.5	69	75	75.5	79	81	83	87	91	101	117	129
Ionic radius ratio (% of Zn)	76.7	778.4	85.2	85.8	89.8	92	94.3	98.8	103.4	114.8	133	146.6

Ratio of their ionic radius with respect to Zn^2+^ ionic radius. The valence state mentioned in the table refers to the one of the doping element in its precursor molecule.

Once the synthesis is completed, the solution is centrifuged to isolate the nanocrystals forming a powder. The latter is washed in water twice and rinsed in ethanol. Eventually, the powder is annealed at 70 °C for 4 hours.

### Characterization methods

The crystalline structure of the nanocrystals has first been probed by means of XRD with a Bruker AXS D8 diffractometer using K_α_ radiation of Cu in the Bragg-Brentano configuration to confirm the presence of wurtzite phase of ZnO nanocrystals as well as to identify the presence of other possible phases. Complementarily, the samples have been analyzed by transmission electron microscopy (TEM JEOL 2010F) in the high resolution mode to further confirm both the crystal structure and the crystal sizes. The chemical composition of the samples has been analyzed using Fourier Transform IR spectroscopy (FTIR Bruker Vertex 80) and Rutherford Backscattering Spectroscopy (RBS). RBS has been performed only on the 5% doped samples, given that the elemental sensitivity of RBS is approximately 1 at%. For the doped samples as well as the undoped ZnO reference sample, photoluminescence (PL) spectra were recorded and PL quantum yields were calculated. Each powder sample was pressed onto an indium foil substrate and was excited with a continuous laser at 266 nm (8 mW, Crylas FQCW 266-10) at room temperature. PL emission was dispersed in a spectrometer (iHR Triax 320 Jobin-Yvon) and detected with a Si CCD camera cooled at liquid nitrogen temperature. PL QY measurements were performed using an integrating sphere according to the protocol developed by de Mello *et al*.^36^. The integrating sphere has been calibrated independently with a calibrated quartz tungsten halogen lamp and CdSe/CdS quantum rods colloidal solution of nominal PL QY of 79%.

## Results

### Structural characterization

**Figure 1:**
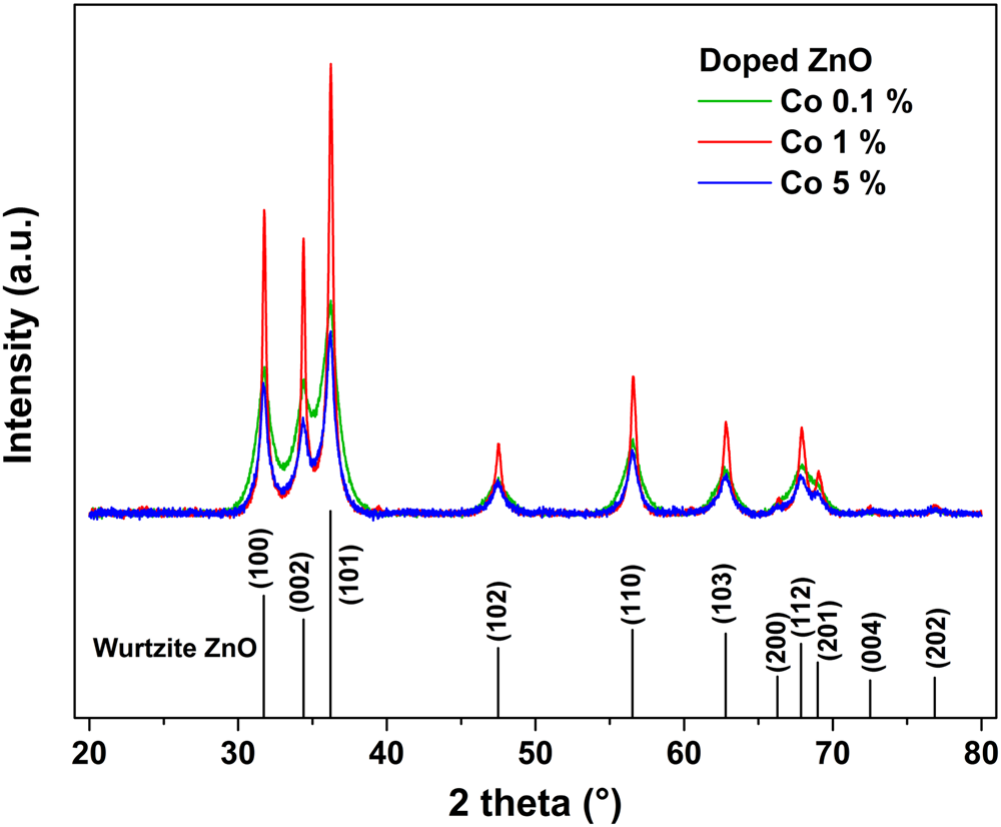
XR diffractograms corresponding to ZnO-PAA nanohybrid samples doped with Co at 0.1%, 1% and 5%. No crystalline phase other than wurtzite ZnO is observed.

XRD has been performed on selected samples. Figure 1 presents the diffractograms of Co doped samples. The diffractograms are representative of the diffractograms obtained from other samples (see Supplementary Information S1).

**Figure 2:**
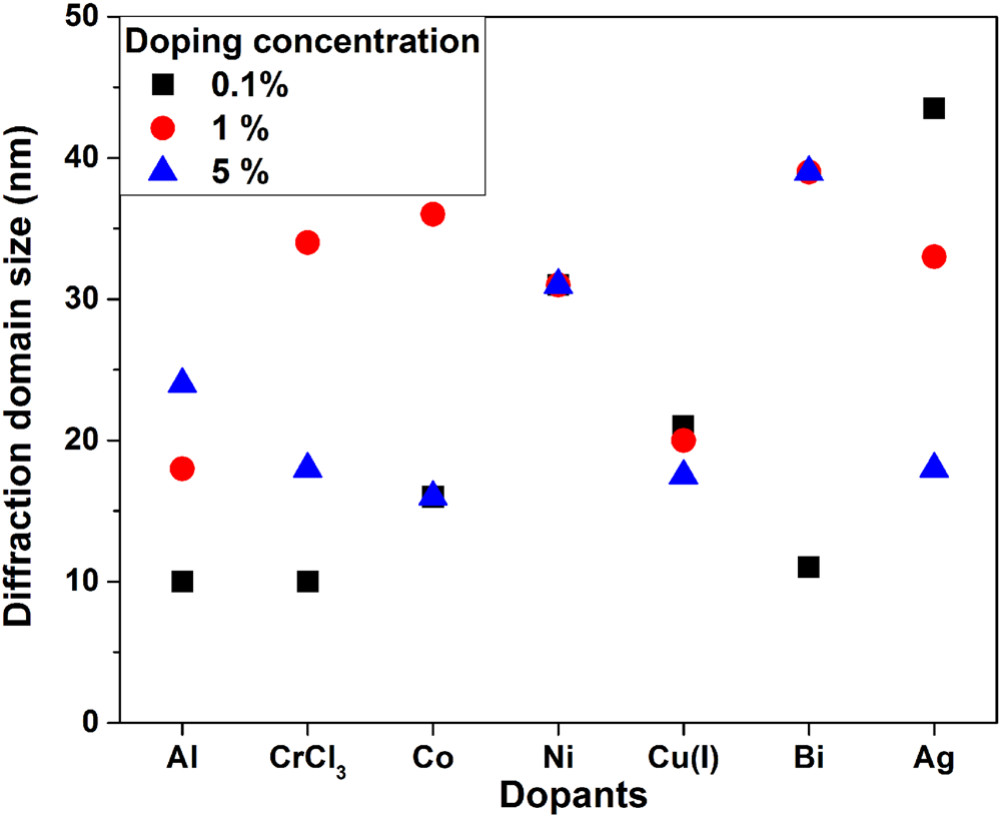
Mean size of the crystalline domains as a function of the dopant nature and concentration.

They confirm the presence of crystallized ZnO in wurtzite phase and the absence of any additional crystalline phase. Using the Scherrer-Debye equation, one can estimate the size of the diffracting domains. Figure 2 presents the results of the calculation of the diffracting domain size as a function of the doping concentration. It appears that the concentration of the doping precursor exhibits no clear trend and most of the time no influence at all on the diffracting domain size, as for Ni. For Ag and Cu(I), the crystal mean size seems to diminish as the doping concentration increases, whereas the contrary happens in the case of Al and Bi doping. Co and Cr do not show a monotonous variation. More significantly, the mean crystal size can vary between 10 and 50 nm under the influence of precursor. This represents a fairly wide range of variation. It is difficult to determine the exact mechanisms responsible for such a large variation. Indeed, several species are in competition during the synthesis process. We established in a previous study that PAAH inhibits the nucleation of ZnO nanocrystals^29^. Here, in addition to the precursor of Zn and PAAH, we introduce dopant precursors in different formulations. These precursors are likely to interact with Zn precursor during the hydrolysis process. This ensures the incorporation of dopants in the obtained samples. However, they are also likely to interact with PAAH. If this is the case, they can hinder the action of PAAH, which leads to a more efficient crystallization process and hence to larger nanocrystals. On the other hand, some could enhance this interaction and lead to smaller crystals. The extent of their interaction with PAAH depends on the complexation power of the dopant precursor, which is not known for all the precursors used in the present study. This point will require further investigation. Besides, we do not notice any significant change in the lattice parameters according to the chemical nature and size of the doping element, as would be expected in the case of a complete substitutional doping.

**Figure 3:**
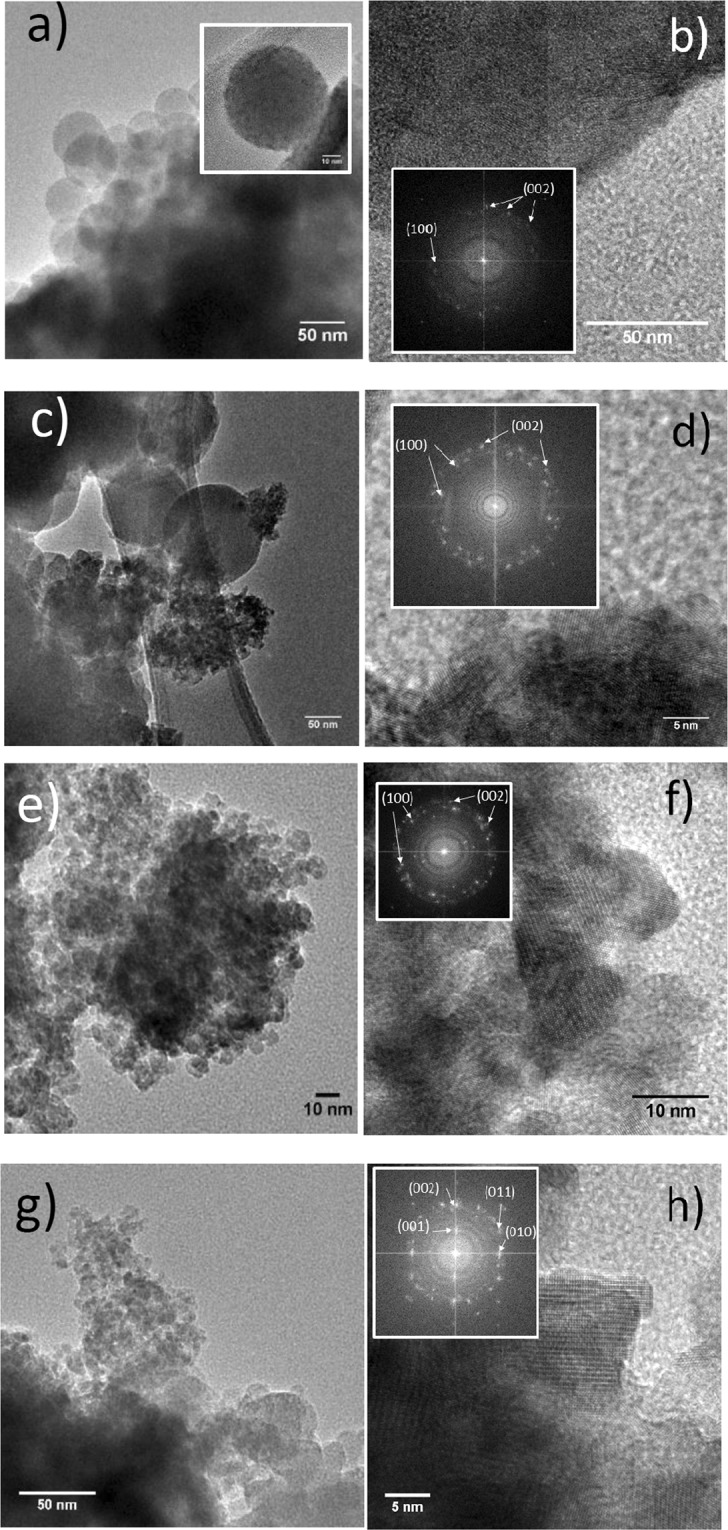
TEM images of ZnO-PAA nanohybrid structures doped with different species: (**a**) Mn 0.1% and (**b**) HRTEM of the same sample. The inset in (**a**) shows the formation of mesospheres of PAA embedding doped ZnO nanocrystals and the inset in (**b**) is the FFT of the image confirming the wurtzite phase of the nanocrystals. (**c**) Mn 5% and (**d**) HRTEM of the same sample with the corresponding FFT (inset). (**e**) Bi 0.1% and (**f**) HRTEM of the sample with the corresponding FFT (inset). (**g**) Cu 0.1% and (**h**) HRTEM of the same sample with the corresponding FFT (inset). Mesospheres are observed at the bottom of image (**g**) while nanocrystals not embedded in PAA are also seen.

Complementary to XRD analysis, we have investigated the nanohybrids using TEM in the high resolution mode. Figure 3 shows images related to samples doped with 0.1% of Cu, Mn and Bi along with the results from the ZnO sample doped with Mn at 5%, to analyze the effect of the precursor concentration. The first observation is that the nature of the dopant precursor has a strong effect on the resulting architecture. For instance, the use of Mn ions in the synthesis leads to the presence of mesospheres made of ZnO nanocrystals embedded in a PAA matrix, as we obtained in a previous study without any dopant^29^. In the mesospheres with a typical size of approximately 50 nm, the nanocrystals have heterogeneous sizes, ranging from 5 nm to more than 10 nm. The case of Cu doping is rather similar. Beside the mesospheres, we can also observe nanocrystals that are not embedded in polymer matrix. In the case of doping with Bi, no polymeric mesosphere is observed. One can clearly distinguish ZnO nanocrystals but these are not embedded in the polymer matrix. No organic-inorganic structure is seen. Eventually, in the case of a doping with Mn at large concentration (5%), the situation is similar to the case of low (0.1%) doping. Mesospheres are still present, but apparently, a larger fraction of nanocrystals is not embedded in them. It is thus clear that the dopant precursor plays a role in the formation of nanohybrids during the synthesis and somehow interacts with the polymeric acid. The high resolution images (Fig. 3b,d,h) and the corresponding FFTs clearly show that ZnO nanocrystals, in the wurtzite structure, are formed in the mesospheres. Since most of these nanocrystals are not independent, it would be highly speculative to estimate an average crystal size from TEM images. The XR diffractograms mentioned above (cf. Fig. 2) are therefore more reliable for estimating the size of the crystalline domain.

### Chemical characterization

In order to answer the question whether the samples contain the doping elements, we performed RBS on selected samples with the highest doping concentration. As explained before, the sensitivity limit of the RBS technique does not allow probing samples with elemental concentrations less or equal to 1 at%. For samples doped with Fe (from Fe^2+^ precursor) and Mn, the measured concentrations were 7.5 ± 0.5% and 7 ± 0.5%, respectively, slightly higher than the value of 5% for the dopant precursor in the solution. However, the analysis confirms the presence of the dopants in the samples. Since the synthesis conditions are generic to all samples, we can assume that the dopants are incorporated in the ZnO nanoparticle samples at any precursor concentration. We also checked that the precursor anion was not incorporated in the samples. For instance, in the RBS spectrum (Supplementary Information S2) corresponding to the Mn doped sample, no trace of Cl^−^ ion was observed, while the dopant precursor was in this case MnCl_2_.

To further confirm that the dopant element is inside the hybrid mesospheres and not segregated apart from them, we performed EDS (energy dispersion spectroscopy) during the TEM analysis on a few isolated mesospheres doped with Mn. The corresponding concentration value was 3.4 ± 1%. This value is somewhat smaller than the RBS one. The discrepancy can result either from the fact that we only performed EDS on a small number of mesospheres with limited statistical meaning or from the existence of segregation of Mn ions in phases outside of the mesospheres. At such a high doping level (7 at%), the second hypothesis is more likely.

**Figure 4:**
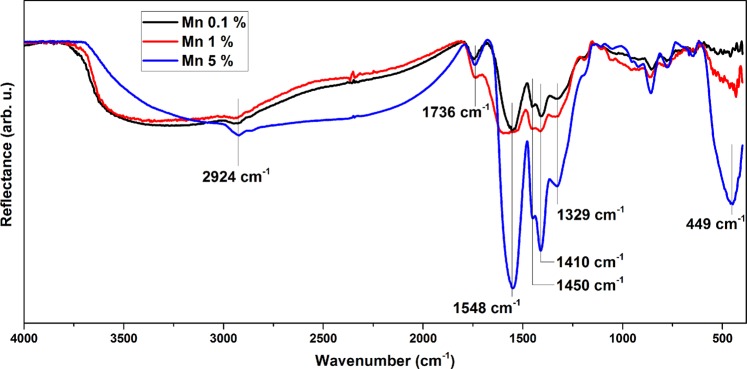
FTIR spectra of Co-doped ZnO nanohybrids with dopant precursor concentrations of 0.1%, 1% and 5% in the solution.

Complementarily, we also performed FTIR study of the doped samples. Figure 4 shows the FTIR spectra of Co doped samples for three distinct precursor concentrations. Other spectra are available in the Supplementary Information (S3). In all samples, the stretching modes of PAA can be observed from 1327 cm^−1^ to 2929 cm^−1^. The two strong peaks at 1547 cm^−1^ and 1406 cm^−1^ are the evidence of the asymmetric and symmetric modes of carboxylate anion (COO^−^), respectively. The broad peak at 442 cm^−1^ can be assigned to Zn-O stretching mode, confirming the presence of ZnO crystallites^37^. For the sample with the smallest dopant precursor concentration, the Zn-O stretching mode is hardly observable revealing the dominating presence of the polymer within the sample. Eventually the broad feature between 3000 and 3500 cm^−1^ is related to water or OH groups.

### Optical characterization

**Figure 5:**
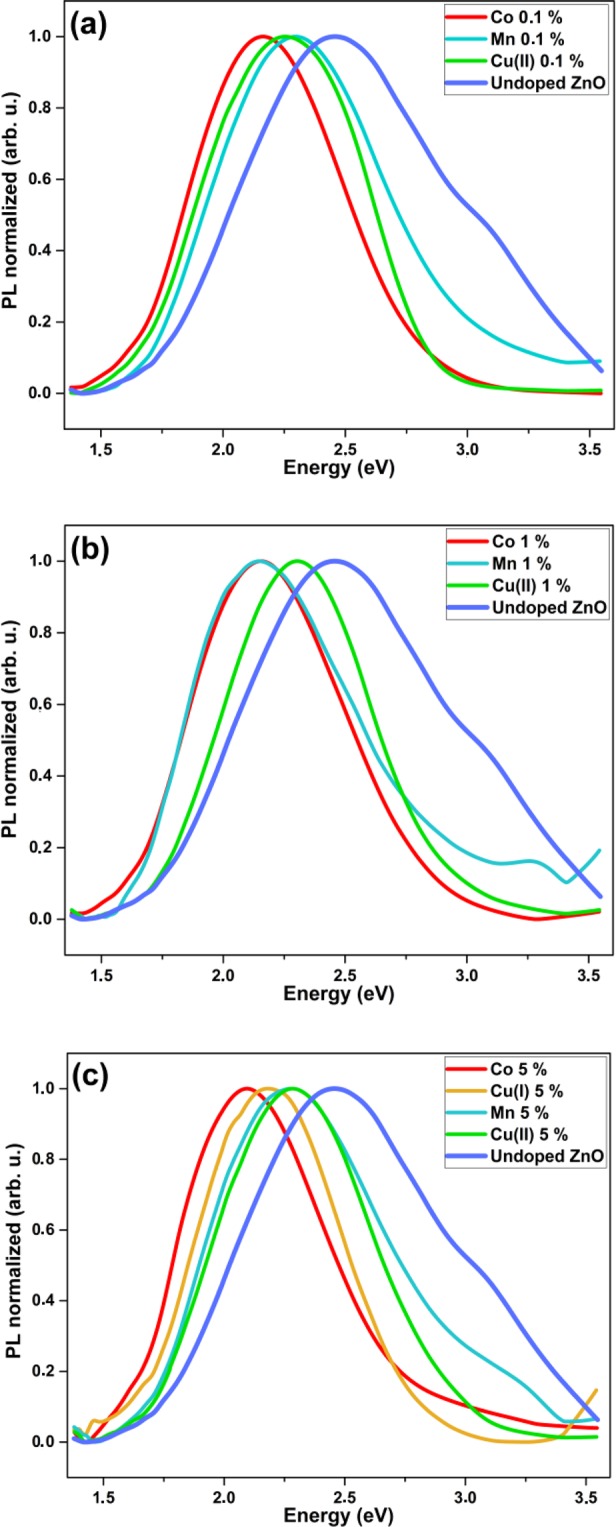
Normalized PL spectra of ZnO nanohybrid samples doped with Co, Cu or Mn at (**a**) 0.1%, (**b**) 1% and (**c**) 5% (dopant precursor concentration). In (**c**), the spectrum of the sample doped with Cu^+^ ions is also shown.

As mentioned above, the main point of the study is to check whether the dopants can modify and help to tune the emission spectrum of ZnO nanohybrids in the visible range without degrading the PL QY. We have thus recorded the PL spectra of all samples (see Supplementary Information S4). Figure 5 shows selected PL spectra of samples doped with Co, Mn and Cu at three distinct concentrations of precursors, namely 0.1%, 1% and 5%. For the latter concentration, on top of the spectrum of the sample doped with Cu ions from Cu^2+^ precursors, the spectrum of the sample doped with Cu ions from Cu^+^ precursors is also plotted to investigate the effect of the precursor valence. It first appears that the spectrum can be tuned in the case of 0.1% doping from 2.46 eV (no dopant) to 2.16 eV (energy of the maximum emission). For the 1% and 5% doping cases, the tuning range spans from 2.46 eV (no dopant) to 2.08 eV. One can notice that, in the 5% doping case, the valence of the Cu ion in the precursor has an effect on the spectrum. Indeed, the spectrum of the Cu^+^ doped sample is red-shifted with respect to that of the Cu^2+^ doped sample. This is not the case for lower doping levels (cf. Supplementary Information S5). It is also worth noting that for most of the samples, near-band-edge UV emission is markedly quenched. This is the result of the significant amount of defect states that compete with the excitonic recombination^25^. The defects inside the nanoparticles corresponding to deep states in the bandgap are therefore the dominant emitting centers.

This tuning ability is only interesting if the PL QY is preserved. Indeed, in many studies published in the literature, a large doping level induces a drastic decrease of the PL QY^17–22^. The visible emission in pure ZnO is composed of several contributions from distinct intrinsic crystalline defects (as oxygen or zinc vacancies or interstitials for instance). The pending question is thus to know whether the given dopant inhibits the emitting defect states of ZnO by introducing quenching states or it induces a peculiar emission related to specific emitting states it introduces into the bandgap.

**Figure 6:**
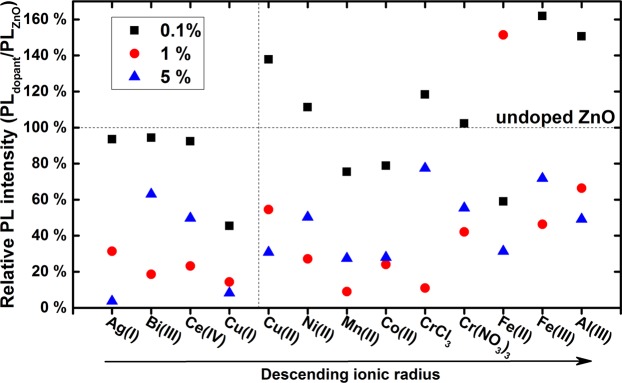
PL QY of doped ZnO nanohybrids normalized to the PL QY of undoped ZnO nanohybrid.

To address this question, it is necessary to accurately measure the PL QY of doped samples and compare it to that of pure (undoped) ZnO nanohybrid. Figure 6 shows the PL QY of all samples normalized to that of pure ZnO nanohybrid sample. The most important point lies in the comparison between the doped and undoped ZnO samples.

The clearest trend is that the larger the dopant concentration, the lower the PL QY. This confirms the observations published in the literature, on systems close to ours. One system does not follow this trend: the samples doped with Fe^2+^ that have the largest PL QY at 1% dopant concentration. This system is at odd with others and remains to be explained.

Moreover and contrary to several studies from the literature, it seems possible for specific dopants to preserve or even enhance the PL QY. This is the case for Ce^4+^, Ag^+^, Bi^3+^, Ni^2+^ (almost identical PL QY) and Cr^3+^, Cu^2+^, Al^3+^ and Fe^3+^ (improvement of the PL QY). In Fig. 6 we have indicated in dashed lines both the normalized PL QY of undoped ZnO nanohybrid and Zn^2+^ ionic radius. Interestingly, the most efficient samples lie in the upper right part. This shows that a small ionic radius and a large valence of the precursor are necessary to improve the PL QY. However, it is not sufficient since some dopants with a 2 + valence and a relatively small ionic radius (Mn and Co) do not improve the PL QY of the ZnO nanohybrids.

## Discussion

**Figure 7:**
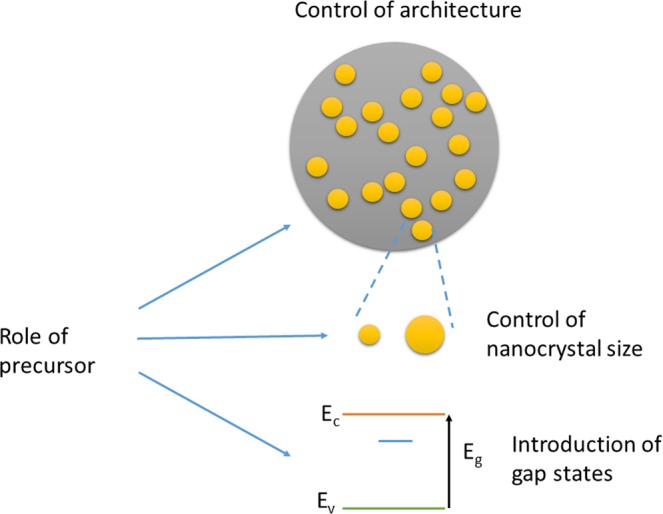
scheme of the effects of the dopant precursor on the resulting ZnO/PAA nanohybrids. The interaction is threefold: affecting the architecture of the mesospheres, of the ZnO nanocrystal size and the amount and nature of the gap states in the ZnO nanocrystals. The latter can be radiative or non-radiative and related to levels of the dopant itself or to native defects of ZnO (vacancies and interstitials).

The dopant plays an important role in tuning the photoluminescence of ZnO nanohybrids. Two major aspects of doping effects are to be considered and summarized. One is the role of dopant in the solution during synthesis, the other is the role of dopant in nanohybrid composite (see Fig. 7).

The doping role during synthesis essentially controls the architecture of sample namely the presence of polymer mesospheres containing ZnO nanocrystals and the size of these nanocrystals.

The interaction between dopant and PAA during synthesis is poorly known, but according to the literature, the function of PAA in solution can be modified by complexing with the dopant to some extent, depending on the chemical nature of the dopant^38,39^. This modifies the pH of the solution and the conformation of PAA chains, leading to the change of interaction between PAA and Zn ions in the solution. However, it is very difficult to explain such multifactorial action of the precursor on resulting nanocrystals since XRD results (Fig. 2) have shown no clear tendency of crystal size evolution induced by dopant precursors. In addition, no further evidence can be provided by FTIR analysis. FTIR analysis mainly confirms the interaction of the deprotonated form of PAA (PAA^−^) with the surface of ZnO nanocrystals (cf. Figure 4 and Supplementary Information S3) but it does not give quantitative information on the fraction of polymer chains interacting with ZnO nanocrystals. The modifications of pH and chain conformation can explain, though, the formation of different morphologies of mesostructures as observed by TEM. Certain dopant species (Bi, for example) forbids the formation of mesosphere structure and high dopant concentration (5% Mn doping, for example) induces larger nanocrystals. The dopant can not only influence the presence or absence of mesospheres, but also the size of the nanocrystals formed in the mesostructures, which probably involves the pH modification of the solution and blocking effect of PAA complexed with dopant ions on crystallization.

With regard to the consequences of architectural change on optical properties, the formation of mesospheres incorporating ZnO nanocrystals has two positive effects. The first one is to improve the light-matter interaction due to mesosphere-induced scattering. It should also help to passivate the surface of the ZnO nanocrystal and thus reduce the presence of quenching surface states. These two effects should therefore improve the quantum efficiency of the PL. Apparently, this effect is not predominant here since mesospheric samples (Cu-doped) have the same PL QY as samples with low mesospheric content (Bi-doped). Control of the size of ZnO nanocrystals is also important since it has been observed that the smaller the nanocrystals, the larger the PL QY is^40^. This trend is roughly observed in our study since large crystal samples all have a low PL QY. For example, in samples doped with Cr ions from the precursor CrCl_3_, the PLQY changes inversely to the crystal size. All the aforementioned effects of the sample architecture affect only the PL QY spectrum and not the PL spectrum. The spectrum is essentially affected by the way dopants are incorporated into the sample and in particular into nanocrystals.

In PAA/ZnO hybrid nanocomposite, the dopants prove to be well incorporated in the mesostructures by RBS and EDS. We can assume that the dopants are incorporated into ZnO nanocrystals, at least partially, introducing electronic levels within the nanocrystal energy diagram. These additional energy levels may be radiative or non-radiative. In the case of Cu^2+^ doping, it has been well documented in the literature that the introduced levels are radiative when Cu ions substitute for Zn ones^23^. For other dopants, however, it is not so clear (see for instance^41^ for Ni doped ZnO where visible luminescence related to oxygen vacancy is induced by the presence of Ni). Since PL QY decreases when the concentration increases, it is obvious that dopants generally introduce non-radiative levels, or quenching levels. It has been reported that Mn and Co occupy the vacancy sites in ZnO nanocrystal, resulting in non-radiative levels^42,43^. These quenching levels are also related to the size of the dopants. Only dopants of comparable or smaller size than Zn tend to maintain or increase PL QY, probably due to better incorporation of dopants into ZnO crystal lattice.

**Figure 8:**
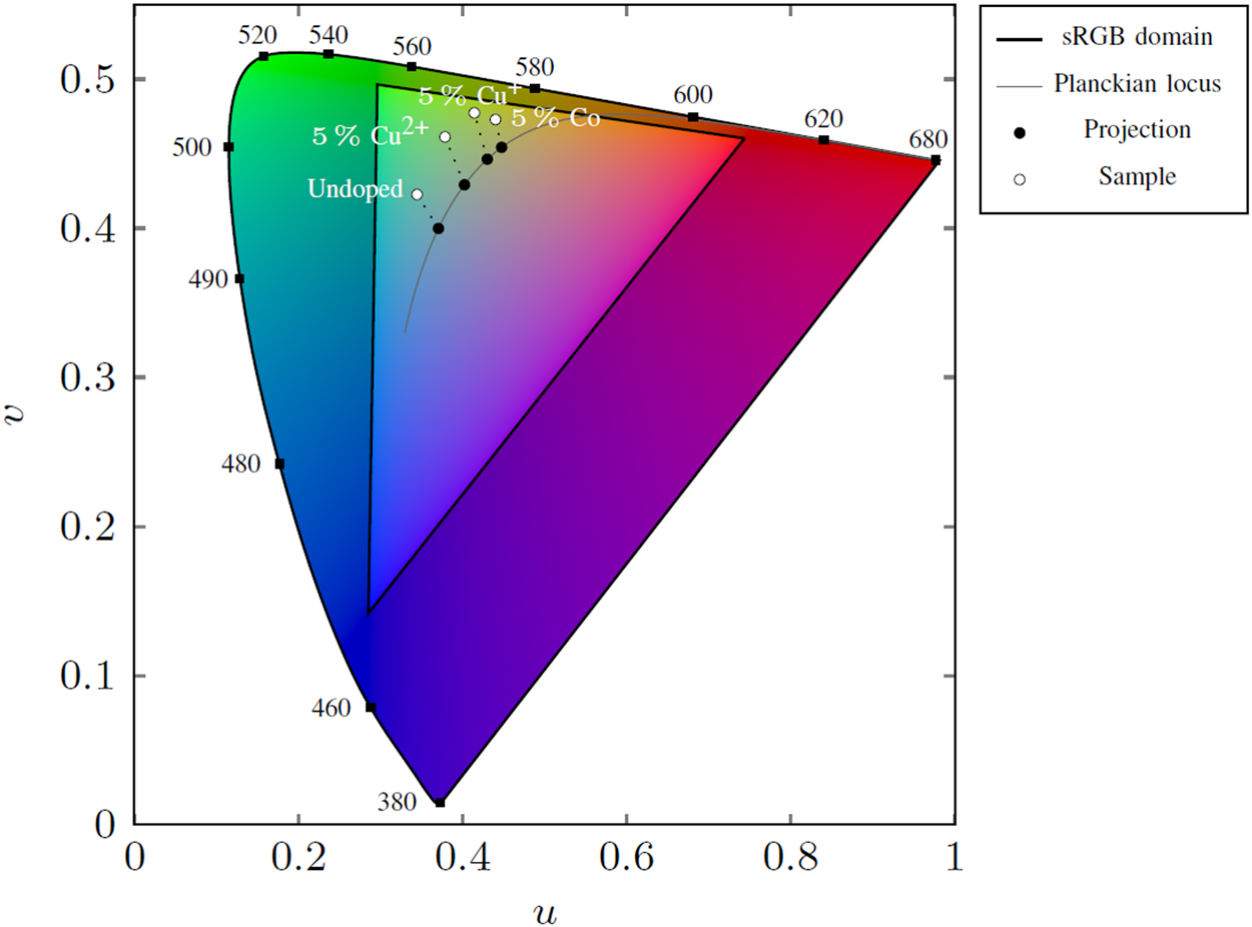
position of the spectra of selected doped samples in the gamut chart in Judd’s UCS diagram.

Among all the doping elements leading to an improvement of PL QY at low doping concentration, namely Cu (from Cu^2+^ precursor), Ni, Cr, Fe (from Fe^3+^ precursor) and Al, only Cu has been found to introduce radiative energy levels in the bandgap [19–21; 30]. For the rest, it is conceivable that their presence favors certain intrinsic radiative defects of ZnO and amplifies their visible photoluminescence. If, on the other hand, each dopant introduces a specific radiative level, then we find these levels varying relatively little even though the dopants are chemically different. The question on whether the dopants introduce radiative levels specific to them remains open and it will be very difficult to decide between the two hypotheses proposed above (inhibition of the emitting defect states of ZnO or introduction of a peculiar emitting states in the ZnO bandgap), even if we favor the first. Nevertheless, from a practical point of view, considering the doping elements leading to an improvement of PL QY at low concentration, it is possible to control the visible emission spectrum between 2.17 eV and 2.47 eV, thanks to the modification of the emission levels. Figure 8 represents on a gamut chart in Judd’s Uniform Chromaticity Scale (UCS) diagram the amplitude of the tuning range effectively allowed. This opens up the prospect of synthesizing phosphors without rare earth element for white LEDs, whose spectrum can be tuned to render warm or cold white light, by a synthesis process with low environmental impact.

## Conclusion

We presented a general and in-depth study of the effect of dopants in hybrid inorganic/organic ZnO/PAA nanocomposites. It is the first comprehensive quantitative study of the effect of dopants on PL QY of ZnO nanoparticles. Our results confirm the trend observed in literature that the increase in dopant concentration leads to quenching of visible luminescence for ZnO nanocrystals obtained by different processes. However, an interesting result is that such degradation of PL QY is not inevitable in our nanocomposites. For some dopants, low doping concentration can improve or at least maintain PL QY, which makes it possible to tune the visible emission spectrum between 2.17 eV and 2.46 eV. This opens up the prospect of synthesizing phosphors without rare earth element for white LEDs, whose spectrum can be tuned to render warm or cold white light, by a synthesis process with low environmental impact. The improvement of PL QY is possible only for dopant ions with a smaller or comparable ionic radius than Zn^2+^. The chemical nature of the dopants and their precursor has no plainly explicable effect on the size of ZnO nanocrystals. Nevertheless, it is probable that the chemical nature of the dopant plays a role during the synthesis of the hybrid nanocomposites, and more particularly during the complexation of PAA and Zn^2+^, ultimately impacting the mesostructure of hybrid inorganic/organic nanocomposites.

## Supplementary information

supplementary info
