# Supplementary material for: Doping of ZnO inorganic-organic nanohybrids with metal elements
Source: Sci Rep. 2019 Aug 16;9:11959. doi: 10.1038/s41598-019-48497-3 (PMC6697676; doi:10.1038/s41598-019-48497-3)
Supplement: Supplementary file 1 — supplementary info [file 41598_2019_48497_MOESM1_ESM.pdf]

Doping of ZnO inorganic-organic nanohybrids with metal elements

Y. Zhang[1], A. Apostoluk[1], C. Theron[2], T. Cornier[2], B. Canut[1], S. Daniele[2], B. Masenelli[1*]

[1]Institut des Nanosciences de Lyon (INL-UMR5270), Université de Lyon, INSA-Lyon, ECL, UCBL, CPE, CNRS, 69621 Villeurbanne, France

[2]Université de Lyon, F-69000 Lyon, France and IRCE Lyon, CNRS, UMR 5256, 69626 Villeurbanne, France
*corresponding author: bruno.masenelli@insa-lyon.fr ; +33 4 72 43 74 72; orchid: 0000-0002-0254-5376

Supplementary information:

S1: XRD spectra of ZnO doped with (a) Al, (b) $CrCl_3$, (c) Ni, (d) Cu^+, (e) Bi, (f) Ag at 0.1 %, 1 % and 5 %, respectively, and with (g) Mn at 0.1 % and 5 %. These spectra confirm that wurtzite ZnO is the only crystallite phase after doping for most dopants at all doping concentrations except for a few cases with large doping ions where $Zn(OH)_2$ is also present.

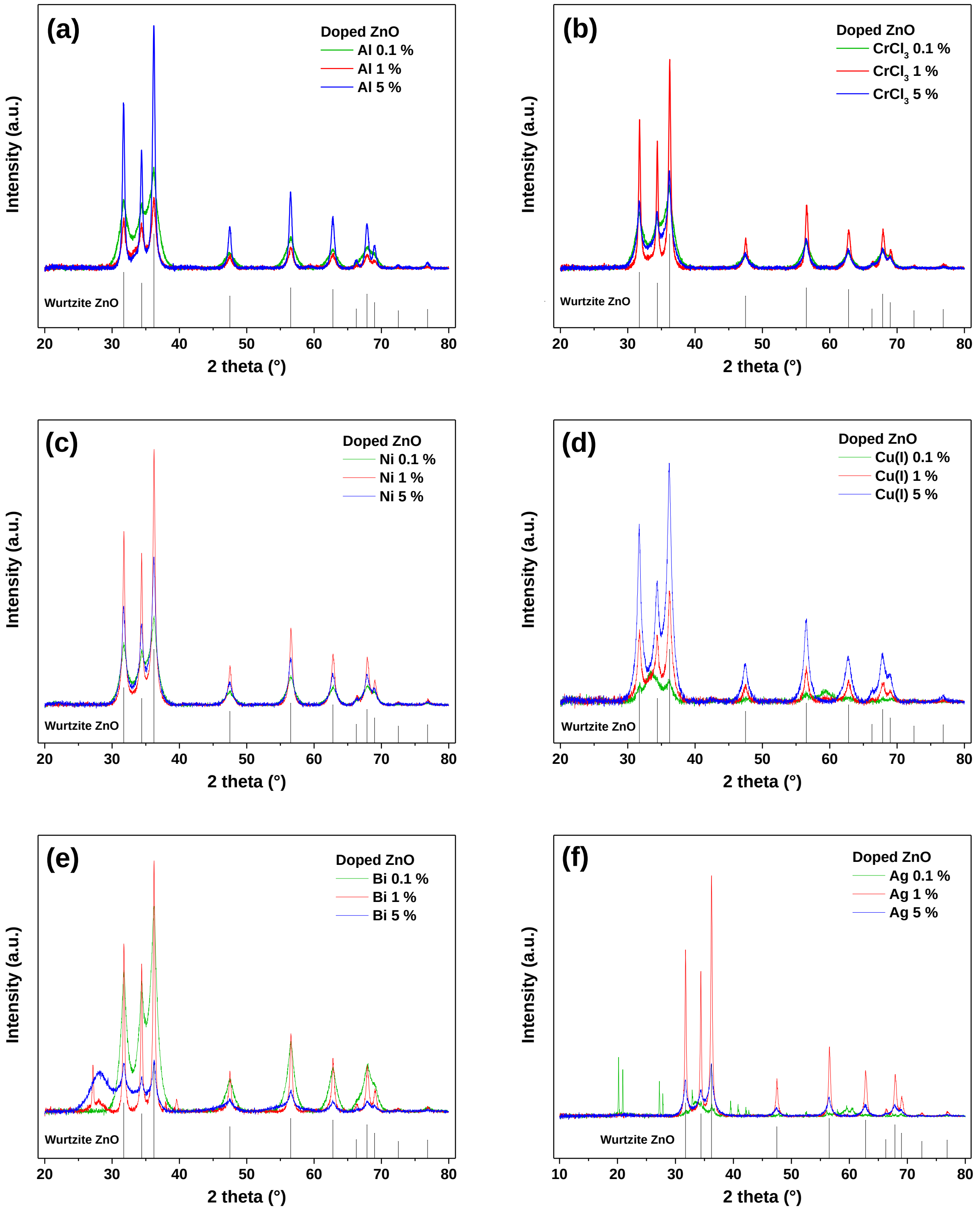

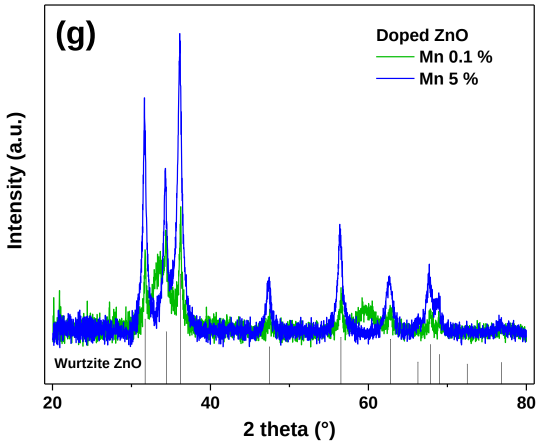
(g)
Doped ZnO
Mn 0.1 %
Mn 5 %
Intensity (a.u.)
Wurtzite ZnO
20
40
60
80
2 theta (°)

S2: RBS of 5 % Mn doped ZnO with simulated curve and contribution of each element. The inset shows detailed contribution from Mn. RBS results prove that Mn is present along with O, Zn and C while no Cl from the precursor is seen.

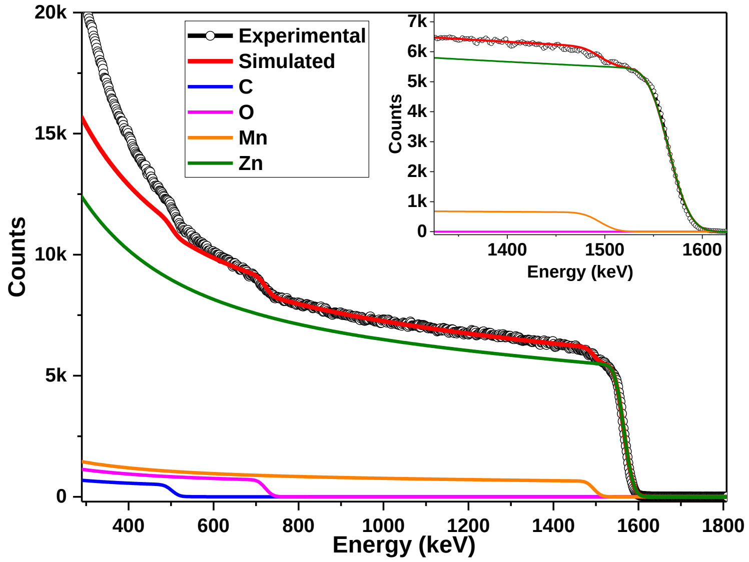

S3: FTIR spectra of doped ZnO at three doping concentrations. For all doping cases, PAA can be distinctively identified by its signature peaks between 1329 cm^{-1} and 2924 cm^{-1} and ZnO can also be identified by **the peak at around 449 cm^{-1}.**

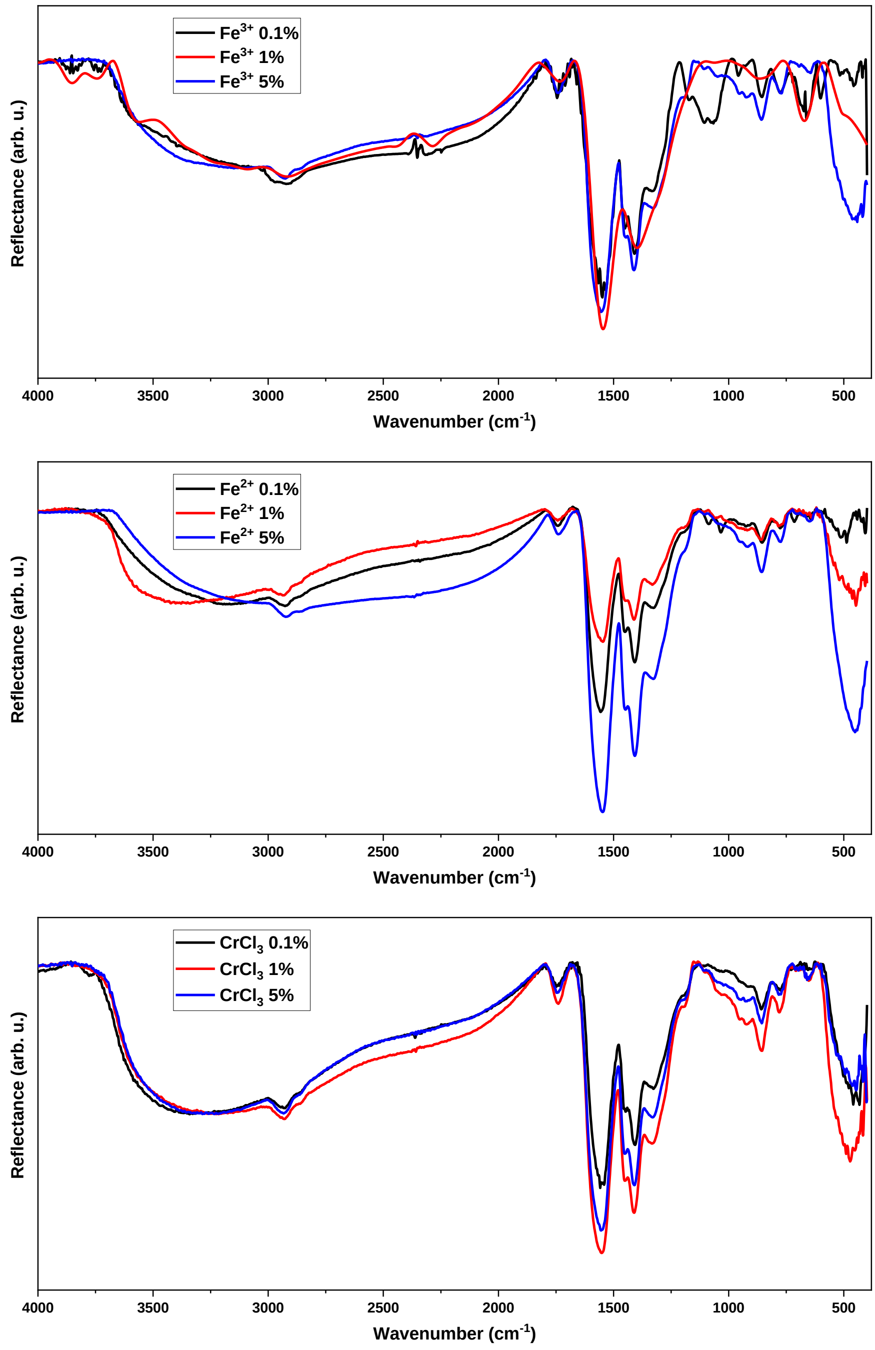

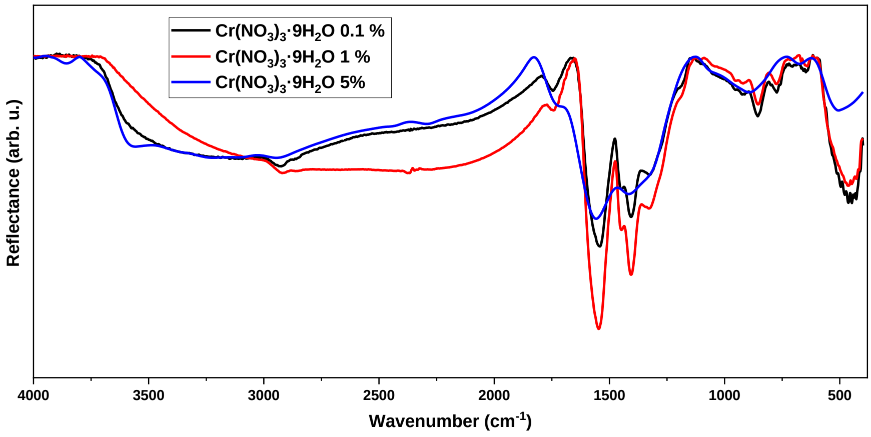

Cr(NO3)3·9H2O 0.1 %
Cr(NO3)3·9H2O 1 %
Cr(NO3)3·9H2O 5%
Reflectance (arb. u.)
4000
3500
3000
2500
2000
1500
1000
500
Wavenumber (cm-1)

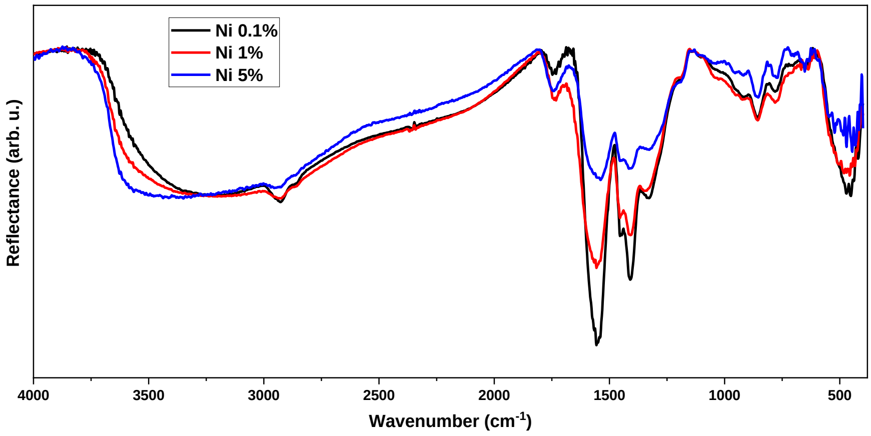

Ni 0.1%
Ni 1%
Ni 5%
Reflectance (arb. u.)
4000
3500
3000
2500
2000
1500
1000
500
Wavenumber (cm-1)

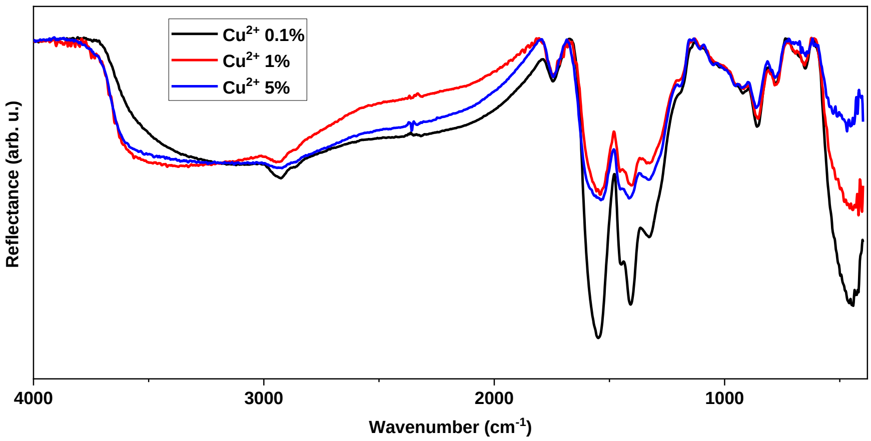

Cu2+ 0.1%
Cu2+ 1%
Cu2+ 5%
Reflectance (arb. u.)
4000
3000
2000
1000
Wavenumber (cm-1)

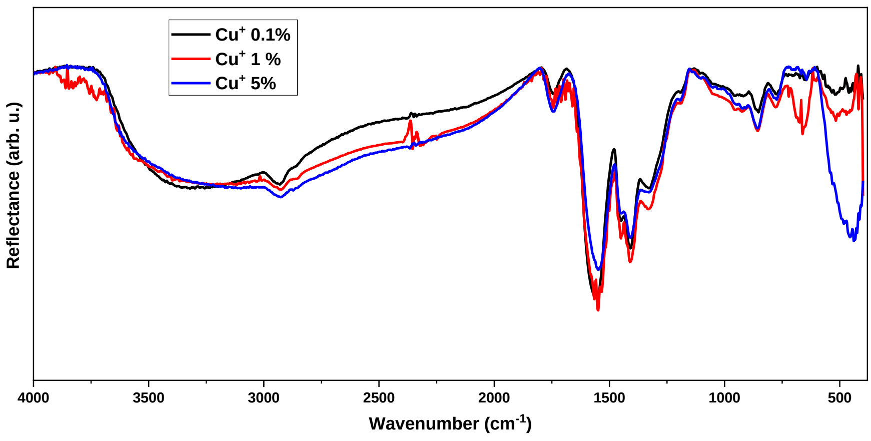

Cu+ 0.1%
Cu+ 1 %
Cu+ 5%
Reflectance (arb. u.)
4000
3500
3000
2500
2000
1500
1000
500
Wavenumber (cm-1)

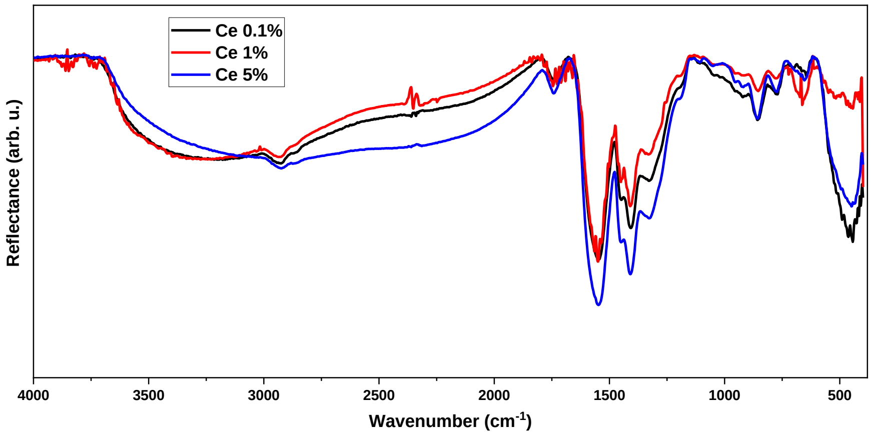
Ce 0.1%
Ce 1%
Ce 5%
Reflectance (arb. u.)
4000
3500
3000
2500
2000
1500
1000
500
Wavenumber (cm-1)

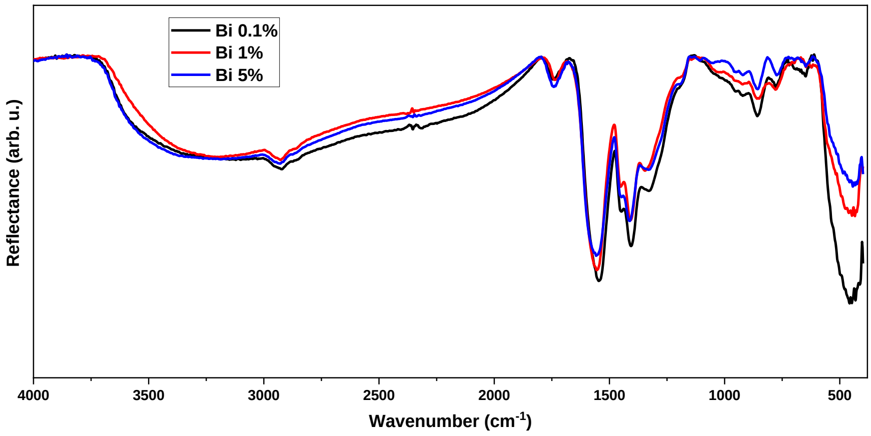
Bi 0.1%
Bi 1%
Bi 5%
Reflectance (arb. u.)
4000
3500
3000
2500
2000
1500
1000
500
Wavenumber (cm-1)

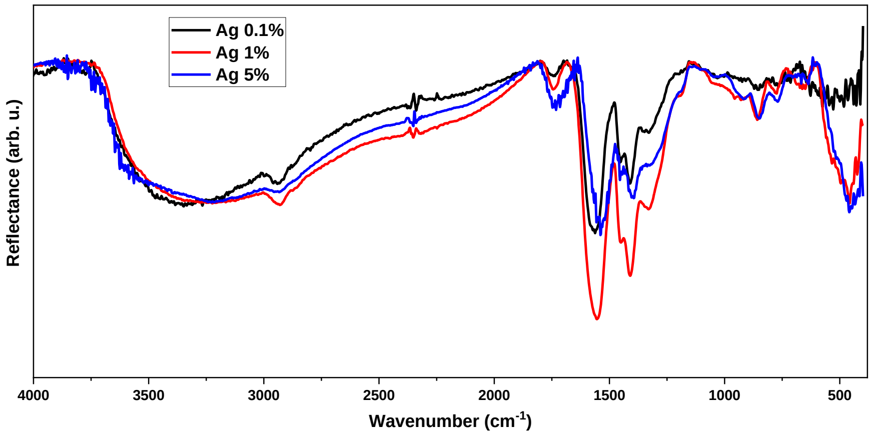
Ag 0.1%
Ag 1%
Ag 5%
Reflectance (arb. u.)
4000
3500
3000
2500
2000
1500
1000
500
Wavenumber (cm-1)

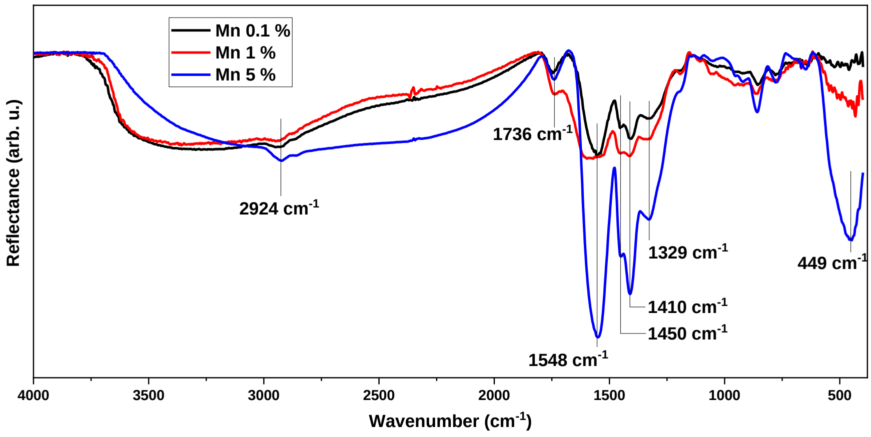
Mn 0.1 %
Mn 1 %
Mn 5 %
Reflectance (arb. u.)
2924 cm-1
1736 cm-1
1548 cm-1
1450 cm-1
1410 cm-1
1329 cm-1
449 cm-1
4000
3500
3000
2500
2000
1500
1000
500
Wavenumber (cm-1)

S4: PL spectra of selected samples

PL spectra of Cr doping with $CrCl_3$ and $Cr(NO_3)_3$ as the precursor at three different concentrations are given in (a), (b) and (c). The shape of the spectra at each concentration with different precursors is the same but the peak positions are slightly different, indicating that the anions, namely, Cl^- and NO_3^-, only have a slight influence on the position of defect levels.

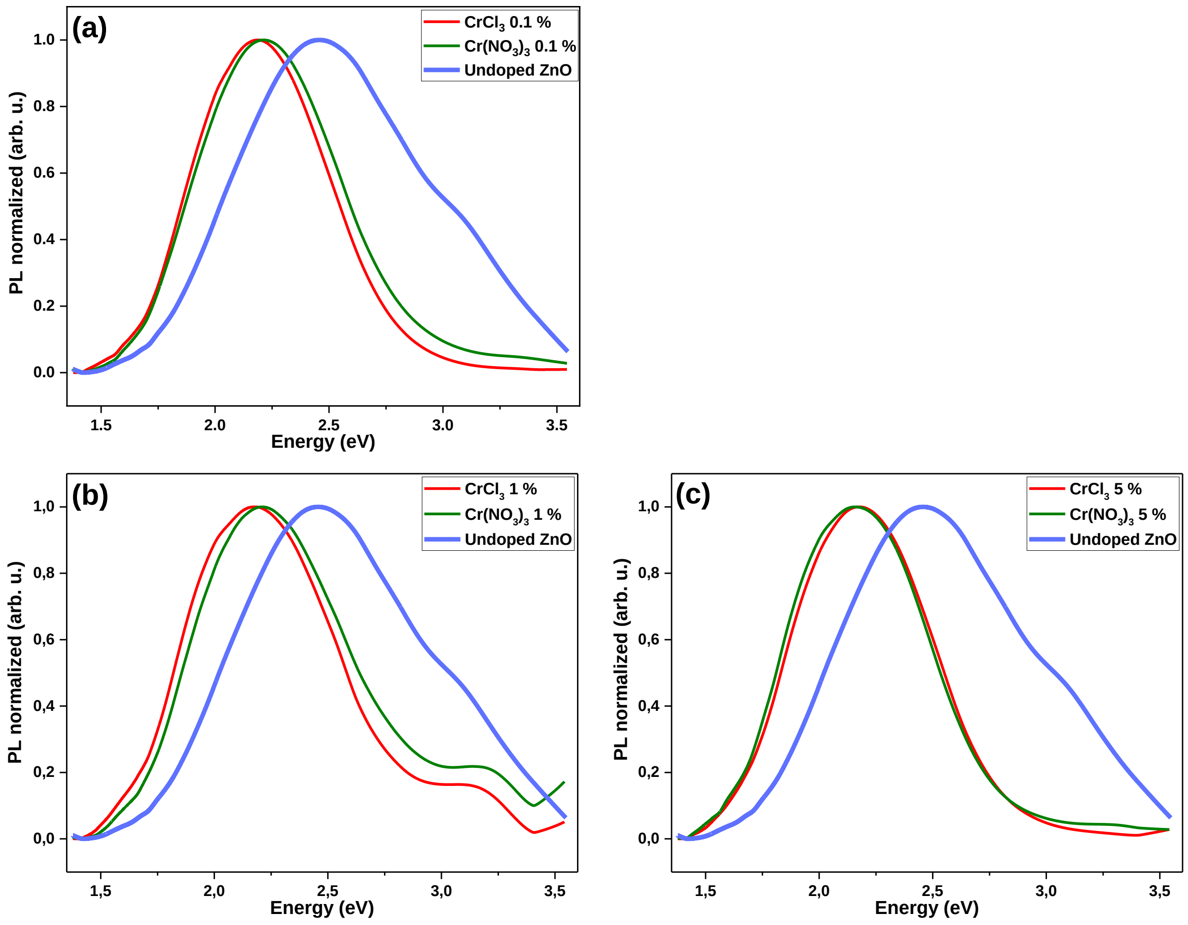

(d) demonstrates the effect of different concentrations of Al doping on the shift of emission peak.

(e) shows the spectra of all samples leading to an increase in the PL QY at 0.1 % doping.

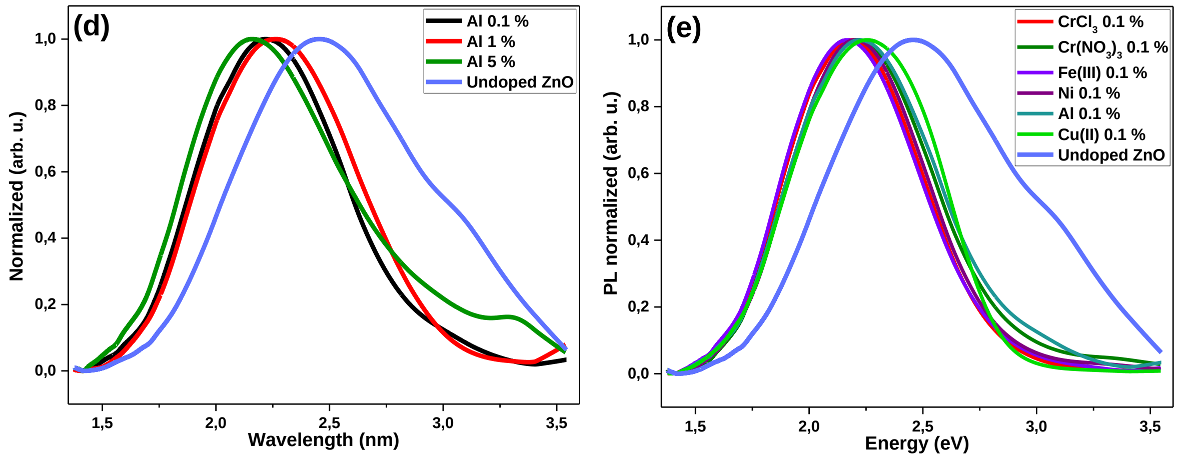

S5: Normalized PL spectra of Cu-doped ZnO/PAA nano-hybrids a) with 0.1%, b) 1% and c) 5% of Cu precursors in the synthesis solution. Cu(I) refers to Cu introduced from precursor with Cu^{+} ions, while C(II) refers to Cu introduced from precursors with Cu^{2+} ions.

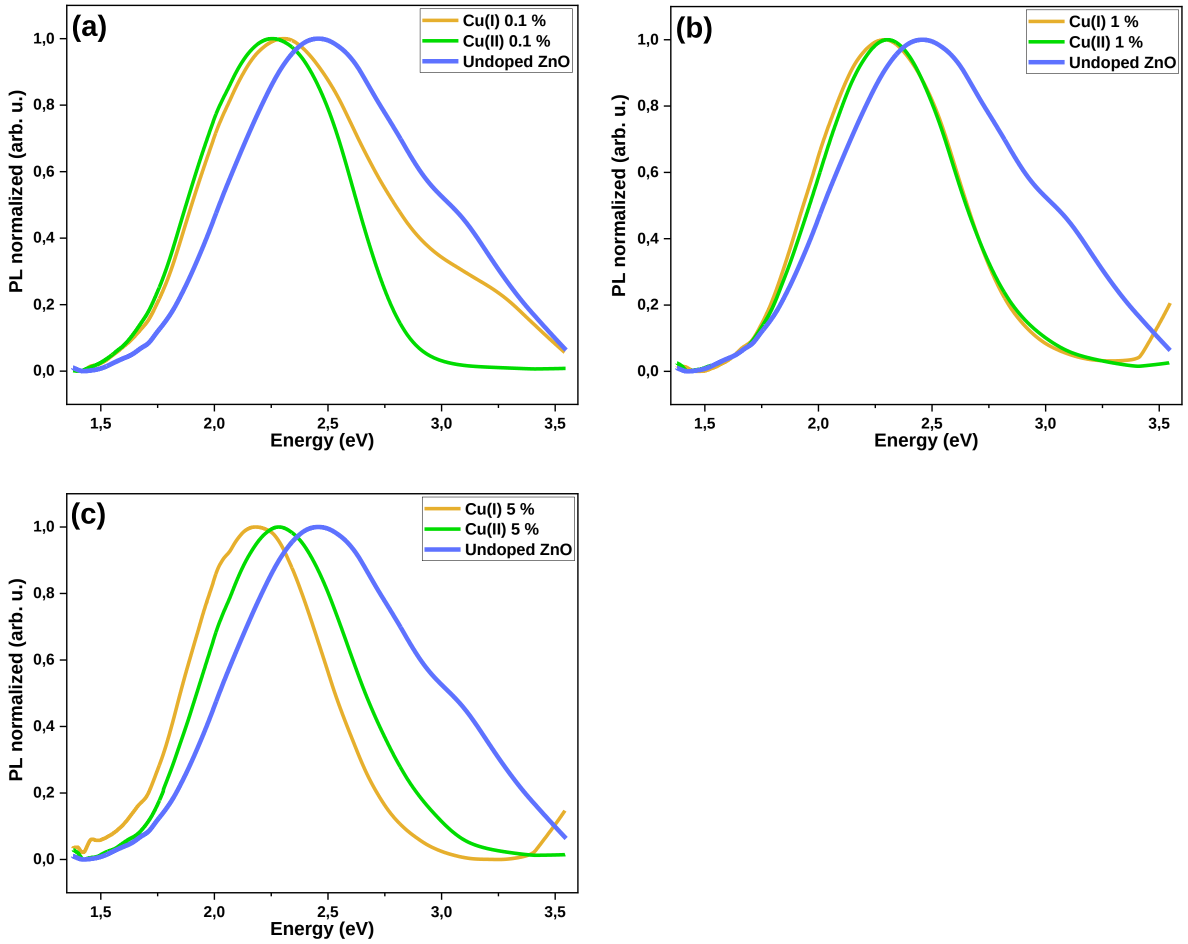

Regarding the spectrum shape, the incorporation of 0.1% or 1% of Cu precursor does not induce significant change in the visible luminescence, whatever the valence of the Cu ion precursor. It goes to show that in both cases, visible emission results from Cu^{2+} gap states. One can notice the band edge UV emission is still present with the use of 0.1% Cu^{+} ion precursor. Concomitantly, the PL quantum yield is reduced when using Cu^{+} ion precursor instead of Cu^{2+} ion precursor. These observations lead us to believe that in both cases, Cu^{2+} ions are present within the ZnO/PAA nanocomposite, giving rise to the well-documented green luminescence. However, in the case of Cu^{+} ion precursor, most of the Cu ions are likely to be inefficiently incorporated (as Cu^{+} ions) regarding the visible luminescence and probably introduce quenching states. For 5% doping level, the spectrum of Cu(I) doped sample is red-shifted with respect to the spectrum of Cu(II) doped sample. At the same time, the PL QY is very weak for both samples but still much smaller for Cu(I) doped sample (cf. figure 6 of main text). It is difficult to establish precisely the nature of the defects induced in ZnO nanohybrids for such a high doping level of Cu^{+} ions. These latter are bound to behave as p-dopant if they substitute Zn ions but they can also behave as n-dopant if they occupy interstitial sites (just as Li ions do). Depending on the type of incorporation, other intrinsic defects (as oxygen vacancies or quenching defects) are introduced. More than the change of PL spectrum, the most important observation is that at 5%, the PL QY drastically drops indicating that quenching states are favored.
